# (5-Methyl­pyrazine-2-carboxyl­ato-κ^2^ *N* ^1^,*O*)bis­[2-(4-methyl­pyridin-2-yl-κ*N*)-3,5-bis­(tri­fluoro­meth­yl)phenyl-κ*C* ^1^]iridium(III) chloro­form hemisolvate

**DOI:** 10.1107/S1600536813034727

**Published:** 2014-01-08

**Authors:** Young-Inn Kim, Young-Kwang Song, Sung Kwon Kang

**Affiliations:** a Department of Chemistry Education and Department of Chemical Materials, Graduate School, Pusan National University, Busan 609-735, Republic of Korea; b Department of Chemistry, Chungnam National University, Daejeon 305-764, Republic of Korea

## Abstract

In the title complex, [Ir(C_14_H_8_F_6_N)_2_(C_6_H_5_N_2_O_2_)]·0.5CHCl_3_, the Ir^III^ atom adopts a distorted octa­hedral geometry, being coordinated by three N atoms (arranged meridionally), two C atoms and one O atom of three bidentate ligands. The complex mol­ecules pack with no specific inter­molecular inter­actions between them. The *SQUEEZE* procedure in *PLATON* [Spek (2009 ▶). *Acta Cryst.* D**65**, 148–155] was used to model a disordered chloro­form solvent mol­ecule; the calculated unit-cell data allow for the presence of half of this mol­ecule in the asymmetric unit.

## Related literature   

For phospho­rescent Ir complexes, see: Chen *et al.* (2010 ▶). For phospho­rescent Ir complexes in OLED, see: Chang *et al.* (2013 ▶); Park *et al.* (2013 ▶); Seo *et al.* (2010 ▶).
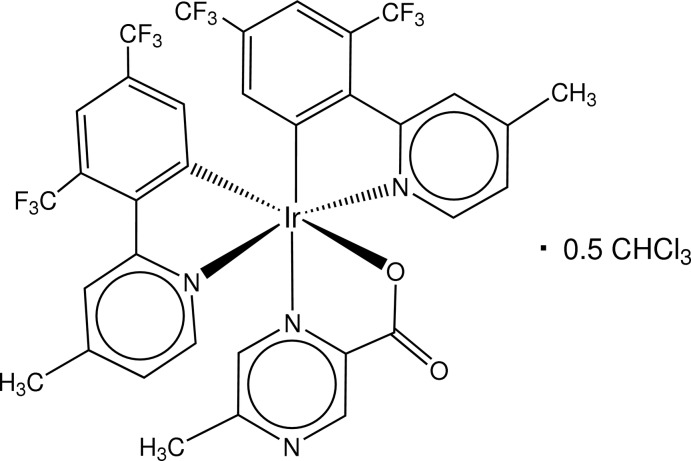


## Experimental   

### 

#### Crystal data  

[Ir(C_14_H_8_F_6_N)_2_(C_6_H_5_N_2_O_2_)]·0.5CHCl_3_
*M* *_r_* = 997.43
Triclinic, 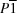

*a* = 11.0949 (3) Å
*b* = 12.3669 (4) Å
*c* = 14.2892 (4) Å
α = 94.399 (3)°
β = 110.888 (1)°
γ = 102.695 (2)°
*V* = 1760.93 (9) Å^3^
*Z* = 2
Mo *K*α radiation
μ = 4.01 mm^−1^
*T* = 296 K
0.36 × 0.27 × 0.26 mm

#### Data collection  

Bruker SMART CCD area-detector diffractometer
Absorption correction: multi-scan (*SADABS*; Bruker, 2002 ▶) *T*_min_ = 0.284, *T*_max_ = 0.351
46539 measured reflections
8709 independent reflections
8016 reflections with *I* > 2σ(*I*)
*R*_int_ = 0.069

#### Refinement  

*R*[*F* ^2^ > 2σ(*F* ^2^)] = 0.025
*wR*(*F* ^2^) = 0.064
*S* = 1.04
8709 reflections
481 parameters
H-atom parameters not refined
Δρ_max_ = 1.22 e Å^−3^
Δρ_min_ = −0.90 e Å^−3^

### 

Data collection: *SMART* (Bruker, 2002 ▶); cell refinement: *SAINT* (Bruker, 2002 ▶); data reduction: *SAINT*; program(s) used to solve structure: *SHELXS2013* (Sheldrick, 2008 ▶); program(s) used to refine structure: *SHELXL2013* (Sheldrick, 2008 ▶); molecular graphics: *ORTEP-3 for Windows* (Farrugia, 2012 ▶); software used to prepare material for publication: *WinGX* (Farrugia, 2012 ▶).

## Supplementary Material

Crystal structure: contains datablock(s) global, I. DOI: 10.1107/S1600536813034727/tk5282sup1.cif

Structure factors: contains datablock(s) I. DOI: 10.1107/S1600536813034727/tk5282Isup2.hkl

CCDC reference:

Additional supporting information:  crystallographic information; 3D view; checkCIF report

## Figures and Tables

**Table 1:** Selected bond lengths (Å)

Ir1—C30	1.993 (3)
Ir1—C9	1.999 (3)
Ir1—N23	2.028 (2)
Ir1—N2	2.035 (2)
Ir1—N44	2.147 (2)
Ir1—O52	2.149 (2)

**Fig. 1.**
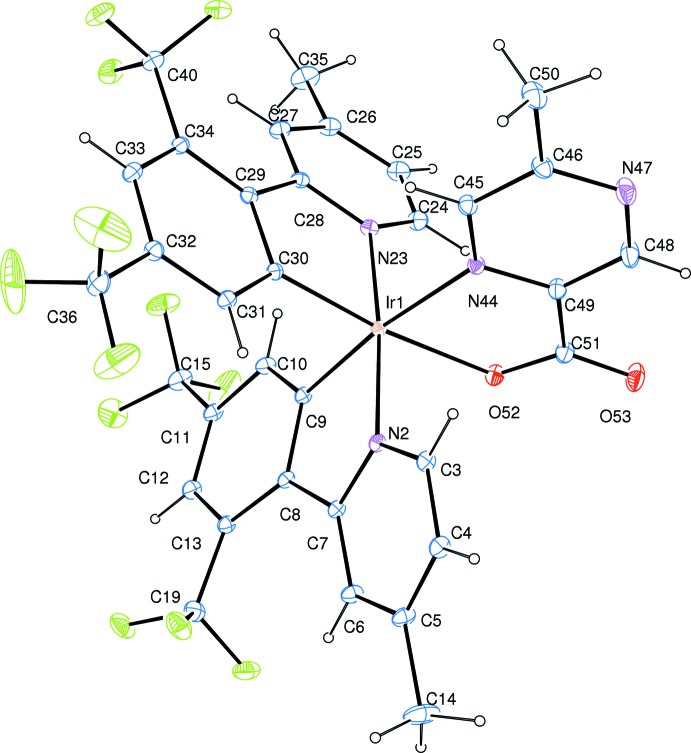
Molecular structure of the title compound, showing the atom-numbering scheme and 30% probability ellipsoids. The chloroform molecule is not shown.

## supplementary crystallographic information

### 2.1. Synthesis and crystallization

Synthesis of 2-(2,4-bis­(tri­fluoro­methyl)­phenyl)-4-methyl­pyridine (dCF_3_pmpy): A Suzuki coupling reaction between 2-bromo-4-methyl­pyridine and 2,4-bis­(tri­fluoro­methyl)­phenyl­boronic acid using tetra­kis(tri­phenyl­phosphine)palladium(0) as a catalyst yielded 2-(2,4-bis­(tri­fluoro­methyl)­phenyl)-4-methyl­pyridine in freshly distilled THF under nitro­gen atmosphere.

Synthesis of title complex: The cyclo­metalated iridium(III) µ-chloro-bridged dimer, [(dCF_3_pmpy)_2_Ir(µ-Cl)]_2_ was prepared from the reaction of the iridium(III) trichloride trihydrate and dCF_3_pmpy in a solution of 2-eth­oxy­ethanol/water (3:1 *v*/*v*). The prepared iridium(III) dimer (0.25 g, 0.15 mmol), sodium carbonate (0.16 g, 1.5 mmol) and 2.2 equivalents 5-methyl­pyrazine-2-carb­oxy­lic acid (mprz) (0.45 g, 0.3 mmol) were dissolved in 2-eth­oxy­ethanol (20 ml) and the mixture was heated at 130 °C for 24 h. The mixture extracted with di­chloro­methane (3 × 50 ml) and dried over anhydrous magnesium sulfate. The crude product was flash chromatographed on silica gel using di­chloro­methane/methanol as an eluent to afford the title iridium(III) complex. Yield: 0.17 g (60%). The yellow crystals were obtained from its n-hexane/chloro­form solution by slow evaporation at room temperature.

### 2.2. Refinement

All H atoms were positioned geometrically and refined using a riding model, with C—H = 0.93-0.96 Å, and with *U*_iso_(H) = 1.2*U*_eq_(C) for aromatic and 1.5*U*_eq_(C) for methyl H atoms. There is a disordered chloro­form solvent molecule which was difficult to model. Therefore, the *SQUEEZE* command of *PLATON* (Spek 2009) was used to model the electron density in the void regions. There is one cavity of 165 Å^3^ per unit cell. This cavity contains approximately 58 electrons which were assigned to one solvent chloro­form (CHCl_3_) molecule. With *Z* = 2, the Ir complex has a 0.5 solvent chloro­form equivalent. The reported molecular formula and derived unit cell characteristics take into account the presence of the solvent molecule. The maximum and minimum residual electron density peaks of 1.21 and -0.90 eÅ^-3^, respectively, were located at 1.16 and 0.84 Å from the F39 and Ir1 atoms, respectively.

### 3. Results and discussion

Phospho­rescent cyclo­metalated iridium(III) complexes have attracted significant attention with respect to their enormous potential in a range of photonic applications (Chen *et al.*, 2010). For example, these iridium(III) complexes can be used as light emitting phosphors in an emitting layer in organic light-emitting diodes (OLEDs) since the emission wavelength of the iridium(III) complexes are tunable from red to blue by changing the electronic nature of the coordinated ligands (Chang *et al.*, 2013; Park *et al.*, 2013; Seo *et al.*, 2010). In this study, we prepared a green emitting Ir(dCF_3_pmpy)_2_(mprz) complex where dCF_3_pmpy is 2-(2,4-bis­(triflouro­methyl)­phenyl)-4-methyl­pyridine and mprz is 5-methyl­pyrazine-2-carb­oxy­lic acid and studied its single-crystal X-ray structure. The title compound showed an emission at 517 nm in a di­chloro­methane solution. The HOMO and LUMO energy levels were obtained -6.04 eV and -3.42 eV from the electrochemical properties, respectively.

In (I), Fig. 1, the Ir^III^ atom is coordinated by three N atoms, two C atoms, and one O atom of three bidentate ligands in a distorted o­cta­hedral geometry. The angles around Ir atoms are in the range of 77.10 (8) – 99.81 (10)°. The Ir—C bond distances of 1.993 (3) – 1.999 (3) Å are shorter than the Ir—N distances of 2.028 (2) – 2.035 (2) Å due to the stronger *trans* influence of the benzene ring compared to the pyridine ring (Table 1). The dihedral angle between the benzene and pyridine rings in the bidentate dCF3pmpy ligands are 16.97 (14) – 16.98 (9)°.

### Crystal data

**Table:**

[Ir(C_14_H_8_F_6_N)_2_(C_6_H_5_N_2_O_2_)]·0.5CHCl_3_	*Z* = 2
*M**_r_* = 997.43	*F*(000) = 966
Triclinic, *P*1	*D*_x_ = 1.881 Mg m^−^^3^
Hall symbol: -P 1	Mo *K*α radiation, λ = 0.71073 Å
*a* = 11.0949 (3) Å	Cell parameters from 9024 reflections
*b* = 12.3669 (4) Å	θ = 2.5–28.3°
*c* = 14.2892 (4) Å	µ = 4.01 mm^−^^1^
α = 94.399 (3)°	*T* = 296 K
β = 110.888 (1)°	Block, yellow
γ = 102.695 (2)°	0.36 × 0.27 × 0.26 mm
*V* = 1760.93 (9) Å^3^	

### Data collection

**Table:**

Bruker SMART CCD area-detector diffractometer	8016 reflections with *I* > 2σ(*I*)
Radiation source: fine-focus sealed tube	*R*_int_ = 0.069
φ and ω scans	θ_max_ = 28.3°, θ_min_ = 2.0°
Absorption correction: multi-scan (*SADABS*; Bruker, 2002)	*h* = −14→14
*T*_min_ = 0.284, *T*_max_ = 0.351	*k* = −16→16
46539 measured reflections	*l* = −19→19
8709 independent reflections	

### Refinement

**Table:**

Refinement on *F*^2^	0 restraints
Least-squares matrix: full	Hydrogen site location: inferred from neighbouring sites
*R*[*F*^2^ > 2σ(*F*^2^)] = 0.025	H-atom parameters not refined
*wR*(*F*^2^) = 0.064	*w* = 1/[σ^2^(*F*_o_^2^) + (0.040*P*)^2^] where *P* = (*F*_o_^2^ + 2*F*_c_^2^)/3
*S* = 1.04	(Δ/σ)_max_ = 0.001
8709 reflections	Δρ_max_ = 1.22 e Å^−^^3^
481 parameters	Δρ_min_ = −0.90 e Å^−^^3^

### Special details

**Table:**

Geometry. All e.s.d.'s (except the e.s.d. in the dihedral angle between two l.s. planes) are estimated using the full covariance matrix. The cell e.s.d.'s are taken into account individually in the estimation of e.s.d.'s in distances, angles and torsion angles; correlations between e.s.d.'s in cell parameters are only used when they are defined by crystal symmetry. An approximate (isotropic) treatment of cell e.s.d.'s is used for estimating e.s.d.'s involving l.s. planes.

### Fractional atomic coordinates and isotropic or equivalent isotropic displacement parameters (Å^2^)

**Table:**

	*x*	*y*	*z*	*U*_iso_*/*U*_eq_	
Ir1	0.03883 (2)	0.25529 (2)	0.20131 (2)	0.02728 (4)	
N2	0.1883 (2)	0.40036 (17)	0.26029 (17)	0.0284 (4)	
C3	0.1693 (3)	0.5036 (2)	0.2518 (2)	0.0333 (6)	
H3	0.0824	0.5112	0.2313	0.04*	
C4	0.2723 (3)	0.5980 (2)	0.2721 (2)	0.0387 (6)	
H4	0.2554	0.6683	0.2681	0.046*	
C5	0.4014 (3)	0.5881 (2)	0.2986 (3)	0.0418 (7)	
C6	0.4208 (3)	0.4820 (2)	0.3097 (2)	0.0383 (6)	
H6	0.5068	0.473	0.3277	0.046*	
C7	0.3165 (3)	0.3897 (2)	0.2949 (2)	0.0290 (5)	
C8	0.3232 (3)	0.2723 (2)	0.3049 (2)	0.0290 (5)	
C9	0.1971 (3)	0.1931 (2)	0.2568 (2)	0.0297 (5)	
C10	0.1925 (3)	0.0790 (2)	0.2549 (2)	0.0358 (6)	
H10	0.1109	0.0255	0.2215	0.043*	
C11	0.3056 (3)	0.0440 (2)	0.3011 (3)	0.0381 (6)	
C12	0.4279 (3)	0.1215 (3)	0.3550 (3)	0.0410 (7)	
H12	0.5031	0.0973	0.3891	0.049*	
C13	0.4371 (3)	0.2356 (2)	0.3577 (2)	0.0349 (6)	
C14	0.5175 (4)	0.6877 (3)	0.3150 (5)	0.0797 (15)	
H14A	0.4921	0.7315	0.2621	0.12*	
H14B	0.5924	0.6618	0.3137	0.12*	
H14C	0.5419	0.7334	0.3798	0.12*	
C15	0.2965 (4)	−0.0789 (3)	0.2947 (3)	0.0525 (9)	
F16	0.1865 (3)	−0.13855 (19)	0.3003 (3)	0.0883 (9)	
F17	0.3047 (5)	−0.1224 (2)	0.2134 (3)	0.1211 (14)	
F18	0.3937 (3)	−0.1016 (2)	0.3709 (3)	0.1000 (10)	
C19	0.5741 (3)	0.3107 (3)	0.4222 (3)	0.0493 (8)	
F20	0.6443 (2)	0.35089 (19)	0.3678 (2)	0.0669 (6)	
F21	0.5699 (2)	0.40044 (19)	0.47936 (17)	0.0694 (6)	
F22	0.6487 (2)	0.2576 (2)	0.4880 (2)	0.0780 (8)	
N23	−0.0887 (2)	0.09914 (19)	0.14990 (18)	0.0316 (5)	
C24	−0.1141 (3)	0.0403 (3)	0.0586 (2)	0.0440 (7)	
H24	−0.0939	0.0788	0.0104	0.053*	
C25	−0.1684 (3)	−0.0741 (3)	0.0334 (3)	0.0475 (8)	
H25	−0.1842	−0.1115	−0.0306	0.057*	
C26	−0.1994 (3)	−0.1336 (3)	0.1039 (3)	0.0447 (7)	
C27	−0.1780 (3)	−0.0718 (2)	0.1968 (3)	0.0411 (7)	
H27	−0.1999	−0.1091	0.245	0.049*	
C28	−0.1243 (3)	0.0451 (2)	0.2193 (2)	0.0318 (5)	
C29	−0.0885 (3)	0.1206 (2)	0.3161 (2)	0.0307 (5)	
C30	−0.0002 (3)	0.2272 (2)	0.3244 (2)	0.0284 (5)	
C31	0.0482 (3)	0.3046 (2)	0.4146 (2)	0.0339 (6)	
H31	0.1077	0.3735	0.4216	0.041*	
C32	0.0082 (3)	0.2796 (2)	0.4939 (2)	0.0373 (6)	
C33	−0.0821 (3)	0.1794 (3)	0.4843 (2)	0.0390 (6)	
H33	−0.1104	0.1649	0.5371	0.047*	
C34	−0.1315 (3)	0.0999 (2)	0.3966 (2)	0.0343 (6)	
C35	−0.2524 (4)	−0.2593 (3)	0.0832 (4)	0.0676 (11)	
H35A	−0.1801	−0.2927	0.1125	0.101*	
H35B	−0.2933	−0.2853	0.0111	0.101*	
H35C	−0.3176	−0.2803	0.1127	0.101*	
C36	0.0617 (4)	0.3628 (3)	0.5896 (3)	0.0562 (9)	
F37	0.1506 (5)	0.4477 (3)	0.5970 (3)	0.173 (2)	
F38	−0.0266 (4)	0.4019 (5)	0.6057 (4)	0.185 (3)	
F39	0.1054 (7)	0.3199 (3)	0.6689 (2)	0.197 (3)	
C40	−0.2349 (3)	−0.0040 (3)	0.3935 (3)	0.0456 (7)	
F41	−0.34179 (19)	−0.03214 (18)	0.30647 (17)	0.0587 (5)	
F42	−0.2824 (2)	0.0087 (2)	0.46604 (18)	0.0708 (7)	
F43	−0.1863 (2)	−0.09515 (17)	0.40656 (19)	0.0639 (6)	
N44	−0.1189 (2)	0.33246 (19)	0.13102 (18)	0.0319 (5)	
C45	−0.2095 (3)	0.3571 (2)	0.1648 (2)	0.0374 (6)	
H45	−0.2079	0.34	0.2274	0.045*	
C46	−0.3056 (3)	0.4073 (3)	0.1089 (2)	0.0435 (7)	
N47	−0.3107 (3)	0.4348 (3)	0.0193 (2)	0.0580 (8)	
C48	−0.2181 (4)	0.4116 (4)	−0.0121 (3)	0.0585 (10)	
H48	−0.2178	0.4315	−0.0735	0.07*	
C49	−0.1226 (3)	0.3600 (3)	0.0412 (2)	0.0397 (6)	
C50	−0.4071 (4)	0.4341 (4)	0.1458 (3)	0.0615 (10)	
H50A	−0.3628	0.4778	0.213	0.092*	
H50B	−0.4663	0.3655	0.1475	0.092*	
H50C	−0.4576	0.4763	0.1009	0.092*	
C51	−0.0206 (3)	0.3327 (3)	0.0033 (2)	0.0431 (7)	
O52	0.0579 (2)	0.28101 (18)	0.05964 (16)	0.0395 (4)	
O53	−0.0184 (3)	0.3611 (3)	−0.0767 (2)	0.0648 (7)	

### Atomic displacement parameters (Å^2^)

**Table:**

	*U*^11^	*U*^22^	*U*^33^	*U*^12^	*U*^13^	*U*^23^
Ir1	0.02945 (6)	0.02304 (6)	0.03234 (6)	0.00983 (4)	0.01330 (4)	0.00644 (4)
N2	0.0359 (11)	0.0205 (9)	0.0327 (11)	0.0110 (8)	0.0149 (9)	0.0077 (8)
C3	0.0389 (14)	0.0251 (12)	0.0409 (15)	0.0156 (11)	0.0162 (12)	0.0088 (11)
C4	0.0483 (16)	0.0229 (12)	0.0493 (17)	0.0149 (12)	0.0201 (14)	0.0088 (12)
C5	0.0453 (16)	0.0235 (13)	0.0612 (19)	0.0071 (11)	0.0263 (15)	0.0099 (12)
C6	0.0369 (14)	0.0271 (13)	0.0560 (18)	0.0100 (11)	0.0222 (13)	0.0093 (12)
C7	0.0353 (13)	0.0233 (11)	0.0343 (13)	0.0112 (10)	0.0178 (11)	0.0069 (10)
C8	0.0340 (13)	0.0240 (11)	0.0355 (13)	0.0107 (10)	0.0185 (11)	0.0081 (10)
C9	0.0336 (13)	0.0249 (12)	0.0361 (14)	0.0117 (10)	0.0168 (11)	0.0088 (10)
C10	0.0370 (14)	0.0227 (12)	0.0522 (17)	0.0102 (10)	0.0209 (13)	0.0079 (11)
C11	0.0422 (15)	0.0260 (13)	0.0557 (18)	0.0140 (11)	0.0255 (14)	0.0138 (12)
C12	0.0397 (15)	0.0357 (15)	0.0559 (19)	0.0194 (12)	0.0201 (14)	0.0181 (13)
C13	0.0361 (14)	0.0310 (13)	0.0416 (15)	0.0125 (11)	0.0165 (12)	0.0113 (11)
C14	0.057 (2)	0.0304 (17)	0.159 (5)	0.0059 (16)	0.053 (3)	0.021 (2)
C15	0.060 (2)	0.0298 (15)	0.085 (3)	0.0231 (14)	0.0397 (19)	0.0184 (16)
F16	0.0793 (17)	0.0376 (11)	0.170 (3)	0.0179 (11)	0.0682 (19)	0.0377 (15)
F17	0.245 (4)	0.0408 (13)	0.141 (3)	0.059 (2)	0.134 (3)	0.0245 (15)
F18	0.098 (2)	0.0503 (14)	0.146 (3)	0.0419 (14)	0.0219 (19)	0.0427 (16)
C19	0.0427 (17)	0.0423 (17)	0.057 (2)	0.0148 (14)	0.0092 (15)	0.0157 (15)
F20	0.0406 (11)	0.0589 (13)	0.1015 (18)	0.0074 (9)	0.0287 (11)	0.0243 (12)
F21	0.0719 (15)	0.0548 (13)	0.0567 (13)	0.0155 (11)	−0.0001 (11)	−0.0062 (10)
F22	0.0555 (13)	0.0632 (14)	0.0864 (17)	0.0184 (11)	−0.0105 (12)	0.0251 (12)
N23	0.0298 (11)	0.0281 (11)	0.0356 (12)	0.0076 (9)	0.0119 (9)	0.0017 (9)
C24	0.0470 (17)	0.0455 (17)	0.0396 (16)	0.0099 (14)	0.0193 (14)	0.0002 (13)
C25	0.0469 (17)	0.0432 (17)	0.0467 (18)	0.0044 (14)	0.0198 (14)	−0.0115 (14)
C26	0.0421 (16)	0.0297 (14)	0.060 (2)	0.0055 (12)	0.0210 (15)	−0.0028 (13)
C27	0.0418 (16)	0.0294 (14)	0.0532 (18)	0.0073 (12)	0.0213 (14)	0.0042 (12)
C28	0.0264 (12)	0.0291 (13)	0.0388 (14)	0.0080 (10)	0.0115 (11)	0.0036 (11)
C29	0.0287 (12)	0.0287 (12)	0.0351 (14)	0.0107 (10)	0.0106 (10)	0.0062 (10)
C30	0.0285 (12)	0.0265 (12)	0.0318 (13)	0.0113 (10)	0.0107 (10)	0.0078 (10)
C31	0.0374 (14)	0.0285 (13)	0.0371 (14)	0.0085 (11)	0.0157 (12)	0.0056 (11)
C32	0.0392 (15)	0.0385 (15)	0.0340 (14)	0.0109 (12)	0.0137 (12)	0.0040 (11)
C33	0.0416 (15)	0.0409 (15)	0.0418 (16)	0.0127 (12)	0.0226 (13)	0.0113 (12)
C34	0.0310 (13)	0.0356 (14)	0.0402 (15)	0.0104 (11)	0.0160 (11)	0.0134 (11)
C35	0.079 (3)	0.0299 (16)	0.091 (3)	0.0001 (17)	0.042 (2)	−0.0109 (17)
C36	0.070 (2)	0.053 (2)	0.0418 (19)	0.0057 (18)	0.0253 (17)	−0.0032 (15)
F37	0.222 (5)	0.134 (3)	0.100 (2)	−0.107 (3)	0.098 (3)	−0.074 (2)
F38	0.127 (3)	0.227 (5)	0.164 (4)	0.057 (3)	0.037 (3)	−0.126 (4)
F39	0.375 (8)	0.101 (3)	0.0358 (15)	0.055 (4)	−0.003 (3)	−0.0066 (16)
C40	0.0473 (17)	0.0408 (16)	0.0519 (19)	0.0063 (13)	0.0257 (15)	0.0100 (14)
F41	0.0373 (10)	0.0578 (12)	0.0718 (14)	−0.0018 (9)	0.0198 (10)	0.0047 (10)
F42	0.0751 (15)	0.0686 (14)	0.0758 (15)	−0.0053 (12)	0.0525 (13)	0.0081 (12)
F43	0.0744 (15)	0.0389 (11)	0.0836 (16)	0.0148 (10)	0.0338 (12)	0.0250 (10)
N44	0.0320 (11)	0.0318 (11)	0.0343 (12)	0.0114 (9)	0.0133 (9)	0.0081 (9)
C45	0.0378 (14)	0.0402 (15)	0.0371 (15)	0.0145 (12)	0.0149 (12)	0.0081 (12)
C46	0.0395 (16)	0.0484 (18)	0.0451 (17)	0.0198 (13)	0.0143 (13)	0.0079 (14)
N47	0.0589 (18)	0.082 (2)	0.0509 (17)	0.0445 (18)	0.0234 (15)	0.0273 (16)
C48	0.064 (2)	0.083 (3)	0.049 (2)	0.044 (2)	0.0262 (18)	0.0326 (19)
C49	0.0426 (16)	0.0434 (16)	0.0371 (15)	0.0166 (13)	0.0158 (13)	0.0119 (12)
C50	0.054 (2)	0.087 (3)	0.059 (2)	0.042 (2)	0.0242 (18)	0.017 (2)
C51	0.0473 (17)	0.0476 (17)	0.0416 (16)	0.0182 (14)	0.0211 (14)	0.0133 (13)
O52	0.0465 (12)	0.0419 (11)	0.0398 (11)	0.0198 (9)	0.0224 (9)	0.0114 (9)
O53	0.0802 (19)	0.094 (2)	0.0504 (14)	0.0460 (16)	0.0411 (14)	0.0379 (14)

### Geometric parameters (Å, º)

**Table:**

Ir1—C30	1.993 (3)	C25—H25	0.93
Ir1—C9	1.999 (3)	C26—C27	1.395 (5)
Ir1—N23	2.028 (2)	C26—C35	1.502 (4)
Ir1—N2	2.035 (2)	C27—C28	1.400 (4)
Ir1—N44	2.147 (2)	C27—H27	0.93
Ir1—O52	2.149 (2)	C28—C29	1.480 (4)
N2—C3	1.347 (3)	C29—C34	1.413 (4)
N2—C7	1.370 (3)	C29—C30	1.427 (4)
C3—C4	1.371 (4)	C30—C31	1.400 (4)
C3—H3	0.93	C31—C32	1.388 (4)
C4—C5	1.381 (4)	C31—H31	0.93
C4—H4	0.93	C32—C33	1.374 (4)
C5—C6	1.388 (4)	C32—C36	1.490 (4)
C5—C14	1.509 (4)	C33—C34	1.388 (4)
C6—C7	1.377 (4)	C33—H33	0.93
C6—H6	0.93	C34—C40	1.508 (4)
C7—C8	1.485 (3)	C35—H35A	0.96
C8—C9	1.414 (4)	C35—H35B	0.96
C8—C13	1.414 (4)	C35—H35C	0.96
C9—C10	1.398 (3)	C36—F37	1.241 (5)
C10—C11	1.376 (4)	C36—F38	1.264 (5)
C10—H10	0.93	C36—F39	1.270 (5)
C11—C12	1.386 (4)	C40—F41	1.332 (4)
C11—C15	1.493 (4)	C40—F42	1.334 (4)
C12—C13	1.389 (4)	C40—F43	1.351 (4)
C12—H12	0.93	N44—C45	1.339 (4)
C13—C19	1.507 (4)	N44—C49	1.342 (4)
C14—H14A	0.96	C45—C46	1.386 (4)
C14—H14B	0.96	C45—H45	0.93
C14—H14C	0.96	C46—N47	1.334 (4)
C15—F17	1.285 (5)	C46—C50	1.489 (5)
C15—F16	1.312 (4)	N47—C48	1.333 (5)
C15—F18	1.330 (5)	C48—C49	1.381 (4)
C19—F20	1.333 (4)	C48—H48	0.93
C19—F22	1.334 (4)	C49—C51	1.504 (4)
C19—F21	1.346 (4)	C50—H50A	0.96
N23—C24	1.347 (4)	C50—H50B	0.96
N23—C28	1.361 (4)	C50—H50C	0.96
C24—C25	1.373 (5)	C51—O53	1.228 (4)
C24—H24	0.93	C51—O52	1.280 (4)
C25—C26	1.387 (5)		
			
C30—Ir1—C9	88.44 (11)	C24—C25—C26	119.6 (3)
C30—Ir1—N23	79.81 (10)	C24—C25—H25	120.2
C9—Ir1—N23	92.01 (10)	C26—C25—H25	120.2
C30—Ir1—N2	99.81 (10)	C25—C26—C27	117.2 (3)
C9—Ir1—N2	79.63 (10)	C25—C26—C35	122.3 (3)
N23—Ir1—N2	171.64 (8)	C27—C26—C35	120.4 (3)
C30—Ir1—N44	97.99 (10)	C26—C27—C28	121.5 (3)
C9—Ir1—N44	173.03 (9)	C26—C27—H27	119.2
N23—Ir1—N44	91.77 (9)	C28—C27—H27	119.2
N2—Ir1—N44	96.54 (9)	N23—C28—C27	119.2 (3)
C30—Ir1—O52	173.79 (9)	N23—C28—C29	112.9 (2)
C9—Ir1—O52	96.65 (9)	C27—C28—C29	127.7 (3)
N23—Ir1—O52	96.41 (9)	C34—C29—C30	118.8 (2)
N2—Ir1—O52	84.65 (9)	C34—C29—C28	128.4 (2)
N44—Ir1—O52	77.10 (8)	C30—C29—C28	112.8 (2)
C3—N2—C7	118.7 (2)	C31—C30—C29	118.9 (2)
C3—N2—Ir1	123.82 (19)	C31—C30—Ir1	125.1 (2)
C7—N2—Ir1	116.56 (16)	C29—C30—Ir1	115.97 (19)
N2—C3—C4	122.8 (3)	C32—C31—C30	120.7 (3)
N2—C3—H3	118.6	C32—C31—H31	119.7
C4—C3—H3	118.6	C30—C31—H31	119.7
C3—C4—C5	119.5 (3)	C33—C32—C31	120.6 (3)
C3—C4—H4	120.2	C33—C32—C36	119.5 (3)
C5—C4—H4	120.2	C31—C32—C36	119.8 (3)
C4—C5—C6	117.3 (3)	C32—C33—C34	120.5 (3)
C4—C5—C14	121.9 (3)	C32—C33—H33	119.7
C6—C5—C14	120.8 (3)	C34—C33—H33	119.7
C7—C6—C5	121.9 (3)	C33—C34—C29	120.3 (3)
C7—C6—H6	119.1	C33—C34—C40	115.4 (3)
C5—C6—H6	119.1	C29—C34—C40	124.3 (3)
N2—C7—C6	119.4 (2)	C26—C35—H35A	109.5
N2—C7—C8	113.0 (2)	C26—C35—H35B	109.5
C6—C7—C8	127.5 (2)	H35A—C35—H35B	109.5
C9—C8—C13	119.7 (2)	C26—C35—H35C	109.5
C9—C8—C7	112.8 (2)	H35A—C35—H35C	109.5
C13—C8—C7	127.4 (2)	H35B—C35—H35C	109.5
C10—C9—C8	117.9 (2)	F37—C36—F38	104.0 (5)
C10—C9—Ir1	125.5 (2)	F37—C36—F39	106.9 (5)
C8—C9—Ir1	116.52 (18)	F38—C36—F39	101.3 (5)
C11—C10—C9	121.6 (3)	F37—C36—C32	116.6 (3)
C11—C10—H10	119.2	F38—C36—C32	113.4 (4)
C9—C10—H10	119.2	F39—C36—C32	113.1 (3)
C10—C11—C12	120.7 (3)	F41—C40—F42	105.4 (3)
C10—C11—C15	119.7 (3)	F41—C40—F43	106.7 (3)
C12—C11—C15	119.6 (3)	F42—C40—F43	105.9 (3)
C11—C12—C13	119.4 (3)	F41—C40—C34	113.2 (3)
C11—C12—H12	120.3	F42—C40—C34	112.4 (3)
C13—C12—H12	120.3	F43—C40—C34	112.8 (3)
C12—C13—C8	120.3 (3)	C45—N44—C49	117.6 (2)
C12—C13—C19	114.0 (3)	C45—N44—Ir1	129.33 (19)
C8—C13—C19	125.7 (2)	C49—N44—Ir1	113.06 (19)
C5—C14—H14A	109.5	N44—C45—C46	121.8 (3)
C5—C14—H14B	109.5	N44—C45—H45	119.1
H14A—C14—H14B	109.5	C46—C45—H45	119.1
C5—C14—H14C	109.5	N47—C46—C45	121.2 (3)
H14A—C14—H14C	109.5	N47—C46—C50	116.8 (3)
H14B—C14—H14C	109.5	C45—C46—C50	121.9 (3)
F17—C15—F16	107.5 (4)	C48—N47—C46	116.1 (3)
F17—C15—F18	105.2 (3)	N47—C48—C49	123.9 (3)
F16—C15—F18	103.8 (3)	N47—C48—H48	118.1
F17—C15—C11	113.1 (3)	C49—C48—H48	118.1
F16—C15—C11	113.5 (3)	N44—C49—C48	119.4 (3)
F18—C15—C11	112.8 (3)	N44—C49—C51	117.7 (3)
F20—C19—F22	106.2 (3)	C48—C49—C51	123.0 (3)
F20—C19—F21	106.5 (3)	C46—C50—H50A	109.5
F22—C19—F21	105.2 (3)	C46—C50—H50B	109.5
F20—C19—C13	113.1 (3)	H50A—C50—H50B	109.5
F22—C19—C13	112.3 (3)	C46—C50—H50C	109.5
F21—C19—C13	112.9 (3)	H50A—C50—H50C	109.5
C24—N23—C28	119.3 (2)	H50B—C50—H50C	109.5
C24—N23—Ir1	122.1 (2)	O53—C51—O52	125.7 (3)
C28—N23—Ir1	116.99 (18)	O53—C51—C49	118.9 (3)
N23—C24—C25	123.0 (3)	O52—C51—C49	115.4 (3)
N23—C24—H24	118.5	C51—O52—Ir1	116.56 (19)
C25—C24—H24	118.5		
